# Endovascular Inferior Vena Cava Filters Placed in Duplicated Inferior Vena Cava Syndrome

**DOI:** 10.7759/cureus.58044

**Published:** 2024-04-11

**Authors:** Rachel Oppenheim, Grace J Kim, Nicole Ramon

**Affiliations:** 1 General Surgery, South Pointe Hospital, Warrensville Heights, USA; 2 Vascular Surgery, South Pointe Hospital, Warrensville Heights, USA

**Keywords:** endovascular procedures, deep vein thrombosis (dvt), congenital vascular disease, inferior vena cava filter (ivcf), duplicated inferior vena cava

## Abstract

Duplicated inferior vena cava (D-IVC) is a relatively rare anatomical anomaly. Clinically, these anomalies are incidentally found on computed tomography (CT) or magnetic resonance imaging (MRI). Lack of pre-operative identification of this congenital malformation can lead to incomplete protection against thromboembolism or hemorrhage. We present a case of a 71-year-old male with a duplicated inferior vena cava who underwent insertion of bilateral inferior vena cava filters for deep vein thrombosis (DVT) management.

## Introduction

Duplicated inferior vena cava is an uncommon anatomical variation that results as a consequence of abnormal development of the cardinal veins that ultimately transform into the inferior vena cava. For a majority of the population with this malformation, it will go undetected without clinical consequence as it does not alter the venous blood return from the lower extremities. However, in individuals who require some type of surgical intervention on their inferior vena cava for reasons other than the inherent nature of this aberration, noting a duplicated inferior vena cava can dictate and change the typical management course. Such is the case when inserting inferior vena cava filters, as they will require additional interventions to protect against thromboembolic disease, as will be noted by our case report.

## Case presentation

A 71-year-old male presented with significant weight loss, weakness, and cachexia over several weeks. His past medical history is most notable for atrial fibrillation, diagnosed over 10 years ago, which was treated pharmacologically using diltiazem without anticoagulation according to his Congestive Heart Failure, Hypertension, Age, Diabetes, Stroke/thromboembolic event, Vascular Disease, and Sex score (CHADS2-VASc score). His score was 0-1 at that time, so he did not require management with anticoagulation. Upon his current presentation, a computed tomography scan of his chest, abdomen, and pelvis revealed a segmental pulmonary embolism in the right upper and bilateral lower lobes as well as diffuse thickening of the distal esophagus extending along the entire stomach, which was suggestive of lymphoma versus adenocarcinoma. In addition, a duplicated inferior vena cava was noted.

The patient was initially started on systemic heparin for treatment of the pulmonary embolisms; however, he subsequently developed large-volume hematemesis with a decrease of 2g/dL in his hemoglobin level. Due to his inability to tolerate anticoagulation, the vascular surgery service was consulted for possible placement of an inferior vena cava filter. Given the patient's overall clinical picture, it was felt that he would benefit from inferior vena cava filter insertion, and he was scheduled for placement the following day.

**Figure 1:**
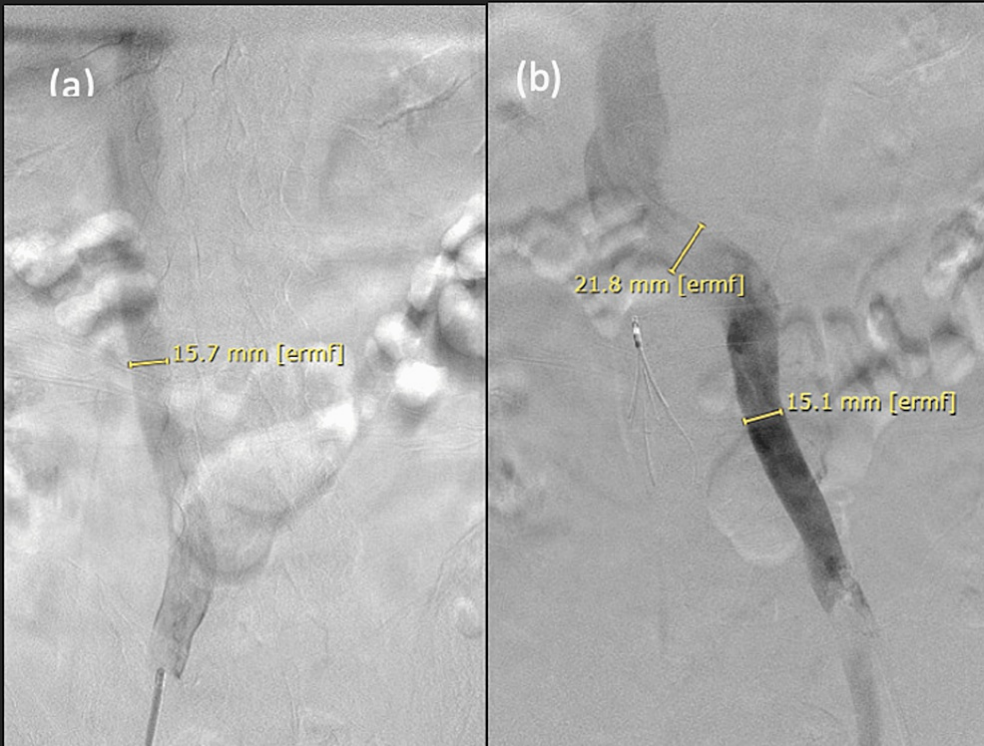
(a) Digital subtraction venogram showing the right and (b) left duplicated inferior vena cava

Prior to venography, the available imaging was reviewed. The computed tomography scan had previously revealed a duplicated inferior vena cava, and a lower extremity ultrasound demonstrated a right peroneal deep vein thrombosis. On a computed tomography scan, the duplicated inferior vena cava appeared to be symmetrical in size, and it was felt that bilateral inferior vena cava filter insertions were a feasible approach. A right-sided approach through the right common femoral vein was first performed, which revealed a patent right inferior vena cava <3 cm in diameter (Figure 1a). The location of the renal veins was confirmed, and an Aln Implants Chirurgicaux® (Bormes-Les-Mimosas, France) inferior vena cava filter was inserted and deployed below the renal veins. Attention was then turned to the left groin, and the left common femoral vein was cannulated. Venography revealed that the left inferior vena cava was patent, <3 cm in diameter, and appeared to converge with the right inferior vena cava supra-renally (Figure 1b). A straight location within the left inferior vena cava was identified, and a second Aln Implants Chirurgicaux® inferior vena cava filter was deployed. The patient tolerated the procedure well without any complications and returned to the intensive care unit post-operatively. He was ultimately transferred to another facility for higher level of care for the management of his inoperable gastroesophageal cancer; however, the patient passed away after his hospital course was complicated by recurrent upper gastrointestinal bleeding, pneumatosis intestinalis, and cardiac arrhythmias.

## Discussion

Our case demonstrates a patient with duplicated inferior vena cava who underwent bilateral inferior vena cava filter placement for management of deep vein thrombosis/pulmonary embolism in the setting of an inability to tolerate anticoagulation secondary to recurrent upper gastrointestinal bleeding. Bilateral Aln Implants Chirurgicaux® inferior vena cava filters were placed in both the right and left inferior vena cava without immediate complications. This is one of three described techniques for the management of an inferior vena cava filter in the setting of duplicated inferior vena cava. However, as duplicated inferior vena cava is a rare occurrence, there is no data on utilizing one technique over another.

Duplicated inferior vena cava is the second most common inferior vena cava abnormality reported, with an incidence of 0.2% to 3% [1,2]. This congenital variance occurs when the primitive cardinal veins come together, and the left supracardinal vein fails to regress during early gestation, resulting in a remnant large vein on both sides of the aorta [2]. These two large veins will independently drain the ipsilateral common iliac veins and form a confluence at, or superior to, where the renal veins would enter the normal anatomical location into the inferior vena cava. 

**Figure 2:**
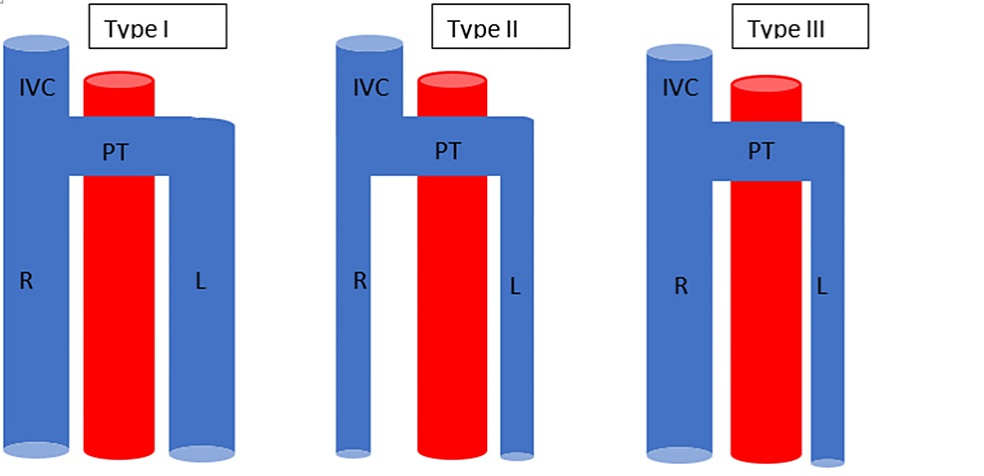
Illustrative representation of the three types of duplicated inferior vena cava IVC - inferior vena cava; R - right inferior vena cava; L - left inferior vena cava; PT - pre-aortic trunk Source: Oppenheim et al. (self-generated)

There are three types of duplicated inferior vena cavas. These are defined based on both the location and the presence of a pre-aortic trunk (Figure 2). Type I, also known as major duplication, is defined as two symmetrically-sized inferior vena cava trunks that are also the same size as the pre-aortic trunk. Type II, or minor type, is defined as two symmetrically-sized inferior vena cava trunks that are smaller in caliber than the pre-aortic trunk. Type III, or asymmetric type, is defined as a thinner left inferior vena cava than the right inferior vena cava with a pre-aortic trunk that is the same size or larger in caliber than the right inferior vena cava [3]. 

Duplicated inferior vena cava can be identified with multiple imaging modalities. Prior to performing an on-table digital subtraction angiography venogram, cross-sectional imaging with multidetector computed tomography or magnetic resonance imaging can be utilized to detect and aid in pre-operative planning when duplicated inferior vena cava is suspected [1,4,5]. Prior studies suggest that computed tomography is the superior imaging modality for inferior vena cava analysis, but it does have limitations. For example, it can be difficult to assess the entire inferior vena cava on a single computed tomography scan as the timing of contrast can only allow for segmental visualization of the inferior vena cava [1].

The indications for inferior vena cava filter placement are similar regardless of inferior vena cava anatomical variation. Three main indications for inferior vena cava filter insertion center around contraindications to treatment of thromboembolic events with anticoagulation: 1) the patient has an absolute contraindication to anticoagulation use, 2) the patient was unable to tolerate anticoagulation/developed a complication from anticoagulation use, or 3) the patient failed anticoagulation therapy [6]. There are also prophylactic indications, with either temporary or permanent filters, in which patients have an increased risk of developing venous thromboembolism but cannot receive anticoagulation, such as in the setting of concomitant traumatic brain injury, prolonged immobilization, and advanced malignancy [6-8].

Once the indication for the inferior vena cava filter has been established and the duplicated inferior vena cava has been identified on imaging, an approach to filter placement must be determined. There are three general approaches: suprarenal inferior vena cava filter, bilateral inferior vena cava filter, or dominant inferior vena cava filter placement with coil embolization of either the non-dominant inferior vena cava or the connecting pre-aortic trunk [5,9]. There is no gold standard approach for inferior vena cava filter insertion in these patients due to the rarity of this anatomical anomaly. The suprarenal approach has been described in duplicated inferior vena cava case reports. However, this technique is not favored by many physicians because of the risk of renal vein compromise with congestion of the filter [10]. The most often described procedure is placement of bilateral inferior vena cava filters, as was performed in our patient [9]. All case reports describing these different approaches had no immediate complications. However, the infrequency of these cases has not allowed for adequate research to evaluate the superiority of one technique over another. Multiple case reports have demonstrated bilateral inferior vena cava filter placement in type I duplicated inferior vena cava [5,9]. In these cases where the right and left inferior vena cavas are the same size as the pre-aortic trunk, it has been assumed that both the right and left inferior vena cavas are of adequate caliber for inferior vena cava filter insertion. Placing bilateral inferior vena cava filters is an advantage over coil embolization because it avoids the complication of ipsilateral lower extremity venous congestion that unilateral coil embolization could potentiate.

In cases where the left inferior vena cava is smaller in diameter than the pre-aortic trunk (type III duplicated inferior vena cava), there was concern that the diameter of the non-dominant inferior vena cava would be inadequate for inferior vena cava filter insertion. So, the non-dominant inferior vena cava was coil embolized while an inferior vena cava filter was placed in the dominant inferior vena cava [9,10]. However, a foreseeable downside to coil embolization is an increased risk of ipsilateral extremity venous congestion, which can lead to complications such as wounds and gait impairment in the long-term setting. To our knowledge, this is the first case report documenting bilateral inferior vena cava filter placement in a patient with type II duplicated inferior vena cava. By demonstrating that inferior vena cava filter insertion was successful and safe in our patient who had minor duplicated inferior vena cavas less than 3 cm, it supports placement of bilateral filter placement in patients with type III duplicated inferior vena cava as well. The insertion of a bilateral inferior vena cava filter is a theoretically favorable option in comparison to inferior vena cava filter insertion with concomitant coil embolization as this allows both lower extremity venous systems to continue draining and reduces the possibility of complications such as lymphedema, although further research is needed.

## Conclusions

Duplicated inferior vena cava is an uncommon anomaly. It can be readily identified on computed tomography imaging and should be further evaluated prior to venography. However, due to the low prevalence of duplicated inferior vena cava, routine computed tomography scans to rule out this anomaly are not recommended. Nonetheless, it is imperative to keep duplicated inferior vena cava as a differential diagnosis when performing procedures, whether they are open or endovascular. This is especially so during venograms because if duplicated inferior vena cava are present and not identified appropriately during placement of an inferior vena cava filter, the patient will have incomplete protection against thromboembolism. While there are three methods of possible inferior vena cava filter placement, no single technique has been proven superior for duplicated inferior vena cava, and the chosen methodology is left to the physician's discretion. Our case report provides evidence that bilateral inferior vena cava filter insertion is a safe and feasible option for multiple sub-types of duplicated inferior vena cavas.
